# DLULIB: a multi-floor WiFi Fingerprinting dataset for indoor positioning in Dalat University library

**DOI:** 10.1016/j.dib.2026.113292

**Published:** 2026-09-29

**Authors:** Khanh Nguyen-Huu, Luong Nguyen Thi, Dang Pham-Hai, Nga Thi Thanh Phan, Trung Vu-Thanh, Seon-Woo Lee, Ninh Duong-Bao

**Affiliations:** a Faculty of Information Technology, Ho Chi Minh City University of Industry and Trade, 700000, Ho Chi Minh City, Vietnam; b Faculty of Information Technology, Dalat University, 670000, Lamdong, Vietnam; c Department of Electronics and Telecommunications, Dalat University, 670000, Lamdong, Vietnam; d College of Computer Science and Electronic Engineering, Hunan University, 410082, Changsha, China; e VNU School of Interdisciplinary Sciences and Arts, Vietnam National University, 100000, Hanoi, Vietnam; f Division of Software, Hallym University, 24252, Chuncheon, Kangwon, Republic of Korea; g Faculty of Mathematics and Computer Science, Dalat University, 670000, Lamdong, Vietnam

**Keywords:** Indoor positioning, WiFi Fingerprinting, Multi-floor dataset, Received signal strength (RSS), University library, Dataset analysis, Positioning benchmark

## Abstract

In this paper a multi-floor WiFi Fingerprinting dataset collected in the library of Dalat University, Vietnam, is presented to support indoor positioning research in complex real-world environments. The dataset was constructed from three floors of the building, which cover open reading areas and corridors under various conditions. Using multiple smartphones operated by different participants and participant-facing directions, a total of 19,500 WiFi fingerprints were collected at 650 reference points (RPs) on three floors. Each fingerprint includes Received Signal Strength (RSS) values collected from 17 access points. It also contains the spatial two-dimensional coordinates of the corresponding RPs and metadata such as device type, participant identifier, and participant facing direction. The dataset includes training (offline phase) and testing (online phase) subsets, which are provided in structured Excel files for each floor. This organization enables flexible use of the dataset for signal analysis, preprocessing evaluation, and algorithm development. Simple-to-complex methods, including k-nearest neighbors, convolutional neural networks, and transformers, are implemented to illustrate dataset usability by considering baseline mean positioning errors. The dataset is publicly accessible through a data repository and can be used for benchmarking indoor positioning approaches, analysis of device heterogeneity, and analysis of signal fluctuation in multi-floor environments.

Specifications Table

**Table:** 

Subject	Computer Sciences
Specific subject area	Indoor positioning using the WiFi Fingerprinting technique and RSS-based positioning
Type of data	Table, Figure, Excel files Raw and Processed data
Data collection	WiFi data were collected using multiple commercial Android smartphones across three floors in the library of Dalat University. The data collection process was conducted at predefined reference points with a grid spacing of approximately 1.2 m. At each point, 20 sets of RSS values were collected from 17 deployed access points under different participants and participant-facing directions.
Data source location	Institution: Dalat University City: Dalat Country: Vietnam Latitude: 11.95536° N Longitude: 108.44412° E
Data accessibility	Repository name: Zenodo Data identification number: 10.5281/zenodo.19434712 Direct URL to data: https://zenodo.org/records/19434712

## 1 Value of the Data

• The dataset addresses the limited availability of public WiFi Fingerprinting datasets from multi-floor buildings in Southeast Asia. DLULIB was collected in a three-floor university library in Vietnam, which offers a realistic and diverse setting for multi-floor indoor positioning.
• The dataset contains 650 RPs, 17 APs, and 19,500 WiFi fingerprints collected under varied device, participant, and orientation conditions, which enables robust evaluation of RSS-based positioning.
• The multi-floor and multi-area coverage provides a realistic setting for evaluating WiFi-based positioning, while spatial coordinates and metadata enable analysis of multi-floor positioning and RSS variations.
• The data are divided into training and testing subsets, with raw and processed files organized in a unified format provided with Python code. These files could be used for the evaluation of preprocessing methods and the development of indoor positioning systems.
• The dataset can be utilized to evaluate various indoor positioning methods, from simple and conventional ones, e.g., k-nearest neighbors (KNN), weighted KNN (WKNN), to complex and data-driven models, e.g., convolutional neural networks (CNNs), transformer-based models, under realistic multi-floor conditions.
• The publicly available Android application and source code facilitate reproducible data collection and adaptation to other indoor environments, which supports the reuse and extension of DLULIB.

## 2 Background

In contrast with the Global Navigation Satellite Systems (GNSS) focusing on outdoor environments, indoor positioning systems (IPSs) aim to track the users/assets’ positions in environments where GNSS is unavailable [1,2]. Various approaches have been applied for IPSs, however, WiFi Fingerprinting has received increasing attention due to its utilization of existing wireless infrastructure and does not require additional hardware deployment [[3], [4], [5]]. In this technique, Received Signal Strength (RSS) values collected at predefined reference points (RPs) will be stored in the fingerprint database and later used to estimate the user’s position.

When evaluating WiFi Fingerprinting-based systems, besides using self-created datasets, research groups can also apply their proposed approaches to public datasets collected in realistic indoor environments. Thus, the performance and generalization of approaches depend strongly on the chosen datasets. Although a number of public datasets have been introduced, many are collected under specific conditions [[6], [7], [8]], such as single-floor layouts or controlled environments, and may not fully reflect the signal fluctuation caused by building structures, user behavior, and device diversity. Furthermore, datasets collected in multi-floor public buildings and Southeast Asian environments remain limited.

As a solution, a new WiFi Fingerprinting-based dataset was created in a multi-floor university library at Dalat University, Vietnam. The dataset includes RSS values collected on different floors, spatial configurations, and usage conditions, along with metadata such as device type, participant ID, and participant-facing direction. The dataset serves as a resource for developing and evaluating indoor positioning methods in complex multi-floor environments. The raw and processed data are stored in an Excel (*.xlsx) file, and some methods are implemented as the baselines for the dataset.

## 3 Data Description

The DLULIB dataset was collected from the library building, as shown in Fig. 1, of Dalat University, Vietnam, which aims to provide one more realistic dataset to support research on WiFi Fingerprinting-based positioning in complex environments. The dataset includes the WiFi RSS data collected on three floors with different spatial areas, such as wide corridors, a central atrium, and open reading areas. These diverse areas, accompanied by related obstacles, human walking and moving, and device diversity, create realistic WiFi signal fluctuations in multi-floor environments. The complete DLULIB dataset is publicly available on Zenodo [9].

**Fig. 1:**
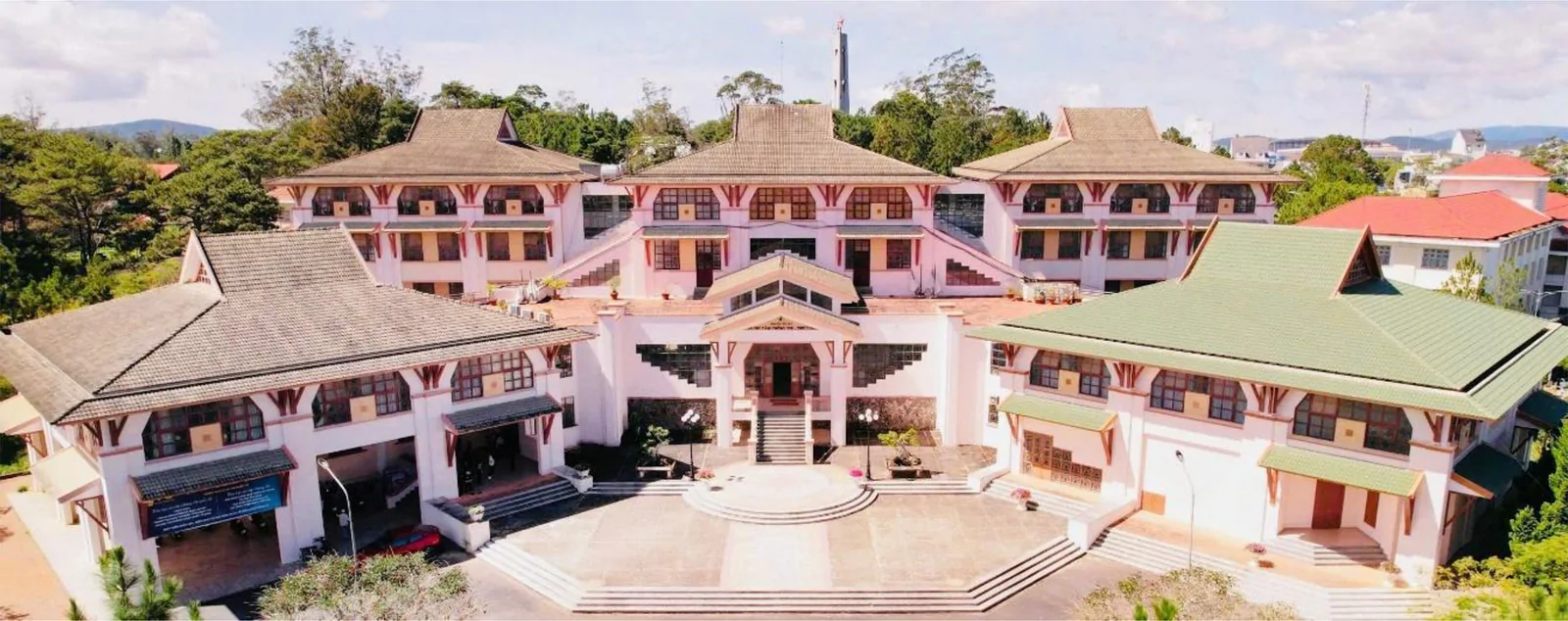
Library building of Dalat University.

The total positioning area in the library building is 966 m^2^, with 482 m^2^ for Floor 1, 268 m^2^ for Floor 2, and 216 m^2^ for Floor 3, respectively. On three floors, a total of 17 WiFi APs and 650 RPs were deployed. The distance between two adjacent RPs is approximately 1.2 m. The number of APs and RPs on the three floors is summarized in Table 1. To be specific, the number of APs deployed on each floor is 7, 6, and 4, and the corresponding number of RPs on each floor is 314, 186, and 150, respectively. Moreover, the spatial distribution of APs and RPs over three floors is presented in Fig. 2. Each red triangle represents an AP, while each grey dot represents an RP. Meanwhile, the orange, blue, green, and pink dots represent RPs and were used as visual markers to help the data collection team verify the recorded RPs and reduce errors such as recording data under an incorrect RP ID.

**Table 1:** Summary of AP and RP deployment in the DLULIB dataset.

Floor	Number of APs	AP labels	Number of RPs	RP labels	RP coordinates	RP spacing
Floor 1	7	AP1 – AP7	314	1 – 314	(6.8, 0.8) – (30.8, 15.2)	1.2 m
Floor 2	6	AP8 – AP13	186	315 – 500	(0.8, 0.8) – (21.2, 14.0)	
Floor 3	4	AP14 – AP17	150	501 – 650	(0.8, 0.8) – (29.6, 6.8)

**Fig. 2:**
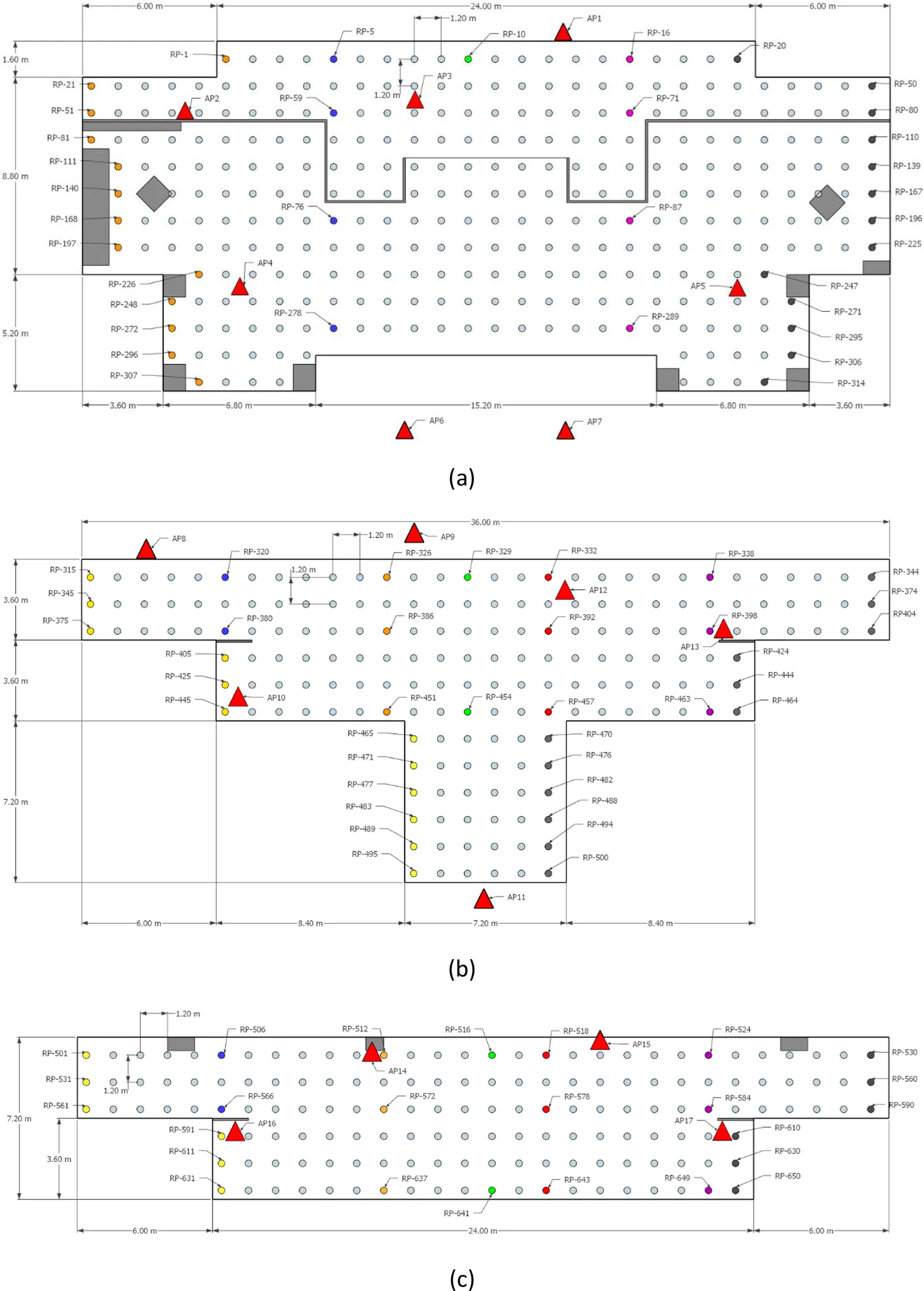
Spatial distributions of APs and RPs in (a) Floor 1, (b) Floor 2, and (c) Floor 3.

The WiFi Fingerprinting technique includes two phases: an offline phase for creating the offline fingerprint database and an online phase for estimating the user’s position. In the DLULIB dataset, sets of RSS values from available APs were first collected at every RP in the former phase to build the fingerprint database, whereas RSS values at test points (TPs) were gathered for positioning evaluation. The DLULIB contains 19,500 fingerprints, including 13,000 fingerprints collected during the offline phase and 6,500 testing samples collected during the online phase. The dataset includes RSS values collected from 17 APs using multiple smartphones, participants, and participant-facing directions. Table 2 summarizes the specified number of fingerprints collected for each floor.

**Table 2:** Summary of collected RSS fingerprints in DLULIB.

Floor	Number of RPs	Fingerprints per RP	Offline fingerprints	Number of TPs	Fingerprints per TP	Online fingerprints	Total fingerprints
Floor 1	314	20	6,280	314	10	3,140	9,420
Floor 2	186	20	3,720	186	10	1,860	5,580
Floor 3	150	20	3,000	150	10	1,500	4,500
Total			13,000			6,500	19,500

The dataset is categorized into three main folders: DLULIB_Floor_1, DLULIB_Floor_2, and DLULIB_Floor_3, as shown in Fig. 3. In each folder, there are three Excel files (*.xlsx), including Training_DLULIB_FloorX_All_20, Training_DLULIB_FloorX_Mean, and Testing_DLULIB_FloorX. The first file shows the whole 20 fingerprints, i.e., sets of RSS values, of each RP, while the second file shows only one representative fingerprint of each RP calculated by averaging 20 fingerprints. The last file includes the RSS values collected at multiple TPs to evaluate the positioning performance. In each file, each row in the dataset corresponds to one fingerprint and includes both the RSS values of the corresponding AP and other metadata, which is summarized in Table 3.

**Fig. 3:**
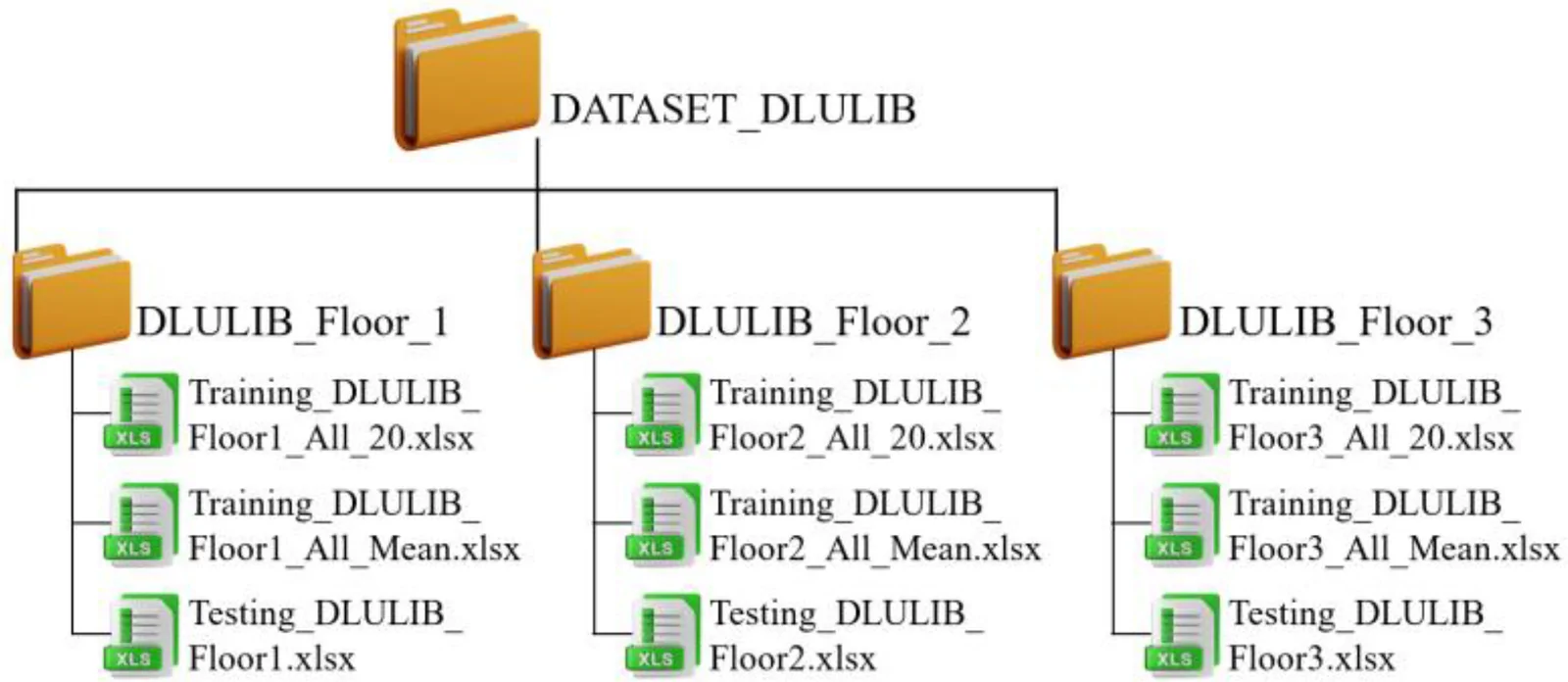
Main folders in the DLULIB dataset.

**Table 3:** Related information in the DLULIB dataset.

Information	Description
ID_RP	Reference point identifier
FloorID	Floor identifier
X, Y (m)	Coordinates of the RP location
AP1-AP17 (dBm)	RSS values collected from 17 APs
Smartphones	Smartphone model used for data collection
Users	Participant identifier
Direction	Participant facing direction (W, N, E, S)
SampleTimes	Collection times at one RP

The dataset reflects fluctuations of the RSS values caused by multiple smartphones, different participants, and four participant-facing directions. The mean RSS distributions of 17 APs for three floors, i.e., Floor 1 to Floor 3, are illustrated in Fig. 4.

**Fig. 4:**
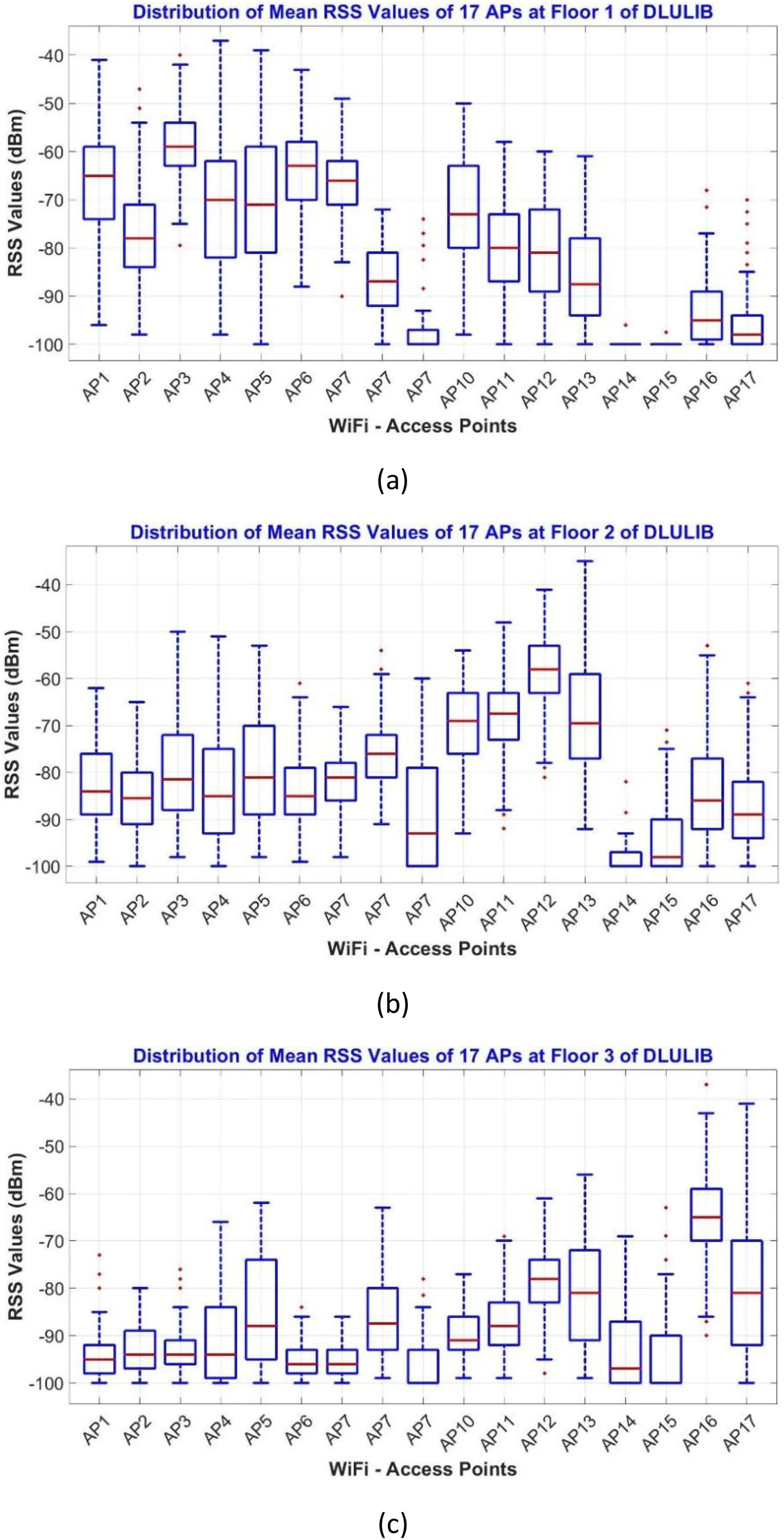
The mean RSS distributions of 17 APs for (a) Floor 1, (b) Floor 2, and (c) Floor 3.

## 4 Experimental Design, Materials, and Methods

### 4.1 Devices and materials

The WiFi data were collected using various Android smartphones with different participants. A variety of smartphone models were considered to reflect signal fluctuation caused by differences in WiFi modules and device hardware. An Android application, called WiFi Data Collection, was built to collect the RSS values from the available APs. The source code of the application is publicly available on GitHub [10]. The application allows participants to input the identifiers of RPs and participants, scan visible APs, and save the collected RSS values into CSV files. The application also saves metadata such as smartphone model and participant-facing directions. Fig. 5 shows the application interface, while Fig. 6 illustrates the workflow of the Android application.

**Fig. 5:**
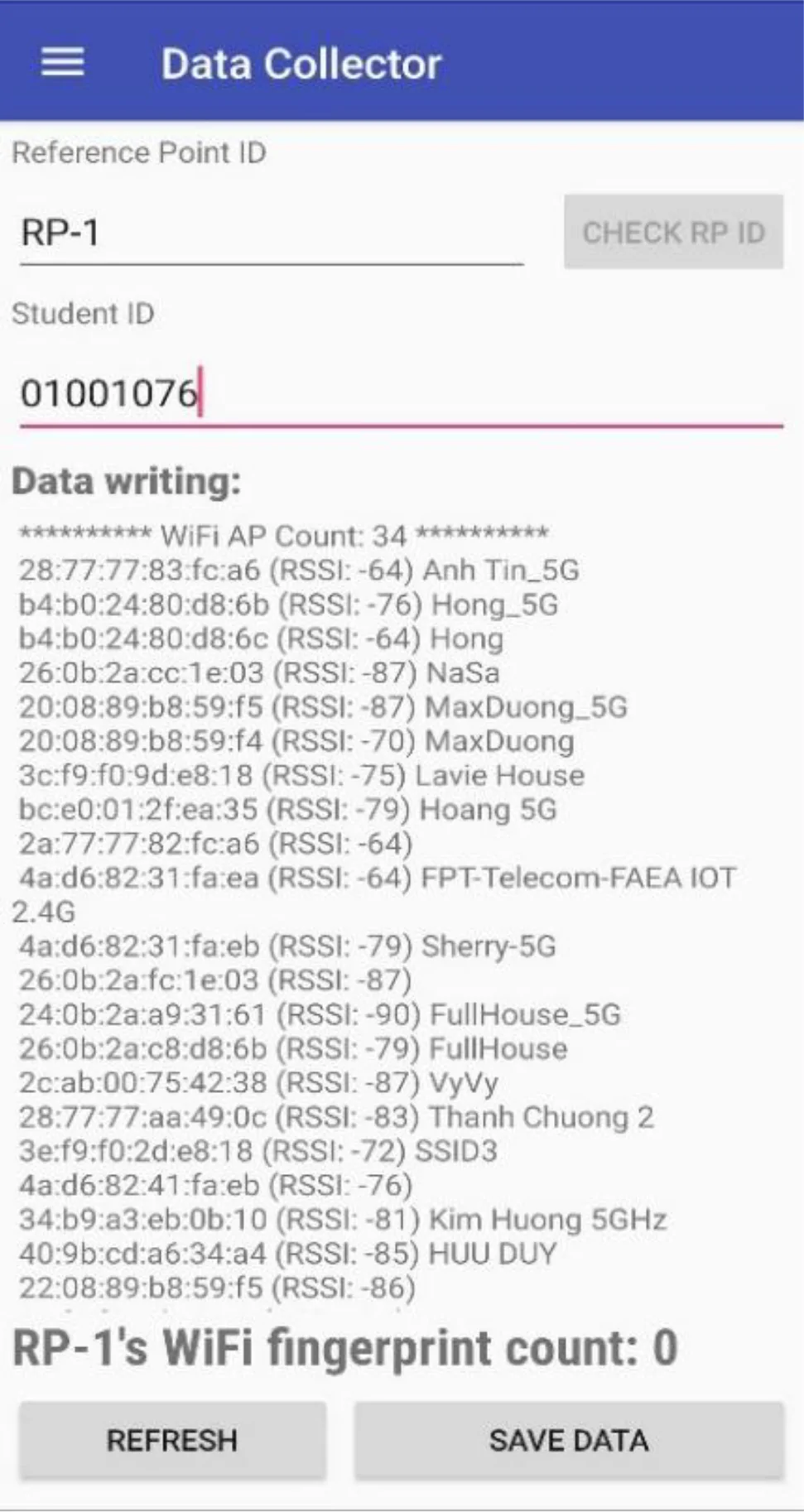
The “WiFi Data Collection” application interface.

**Fig. 6:**
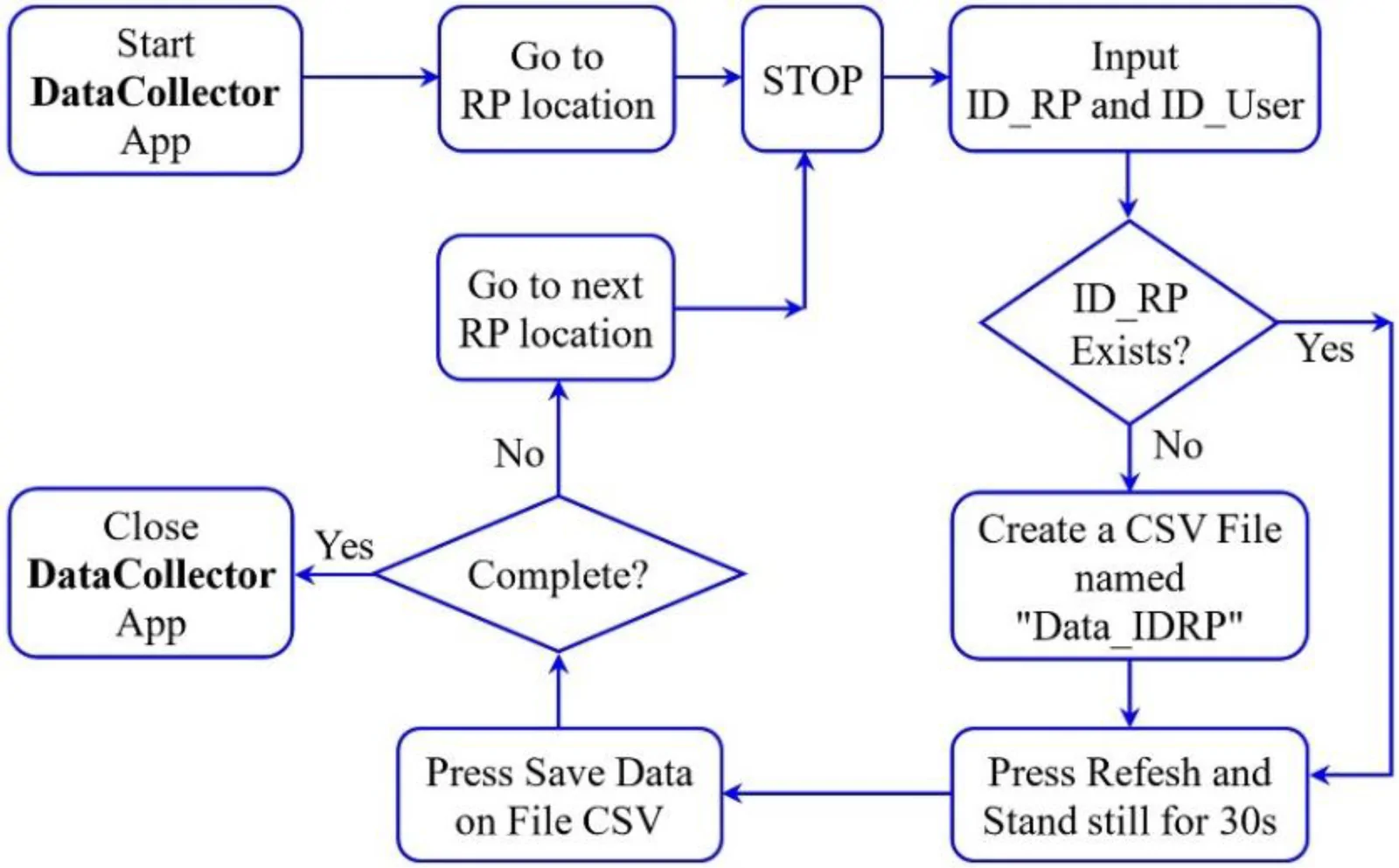
The workflow of the “WiFi Data Collection” application.

### 4.2 Data collection procedure

The data collection process followed two phases of the WiFi Fingerprinting technique. In the offline phase, the RSS values were collected at predefined RPs in four participant-facing directions, including East, West, South, and North. At each RP, the RSS values from available APs were collected five times for each direction, which means that the WiFi data was collected 20 times for each RP in the offline phase. These values were recorded to create the fingerprint database. Fig. 7 illustrates the RSS distribution for a single AP over the RPs on three floors of the DLULIB dataset.

**Fig. 7:**
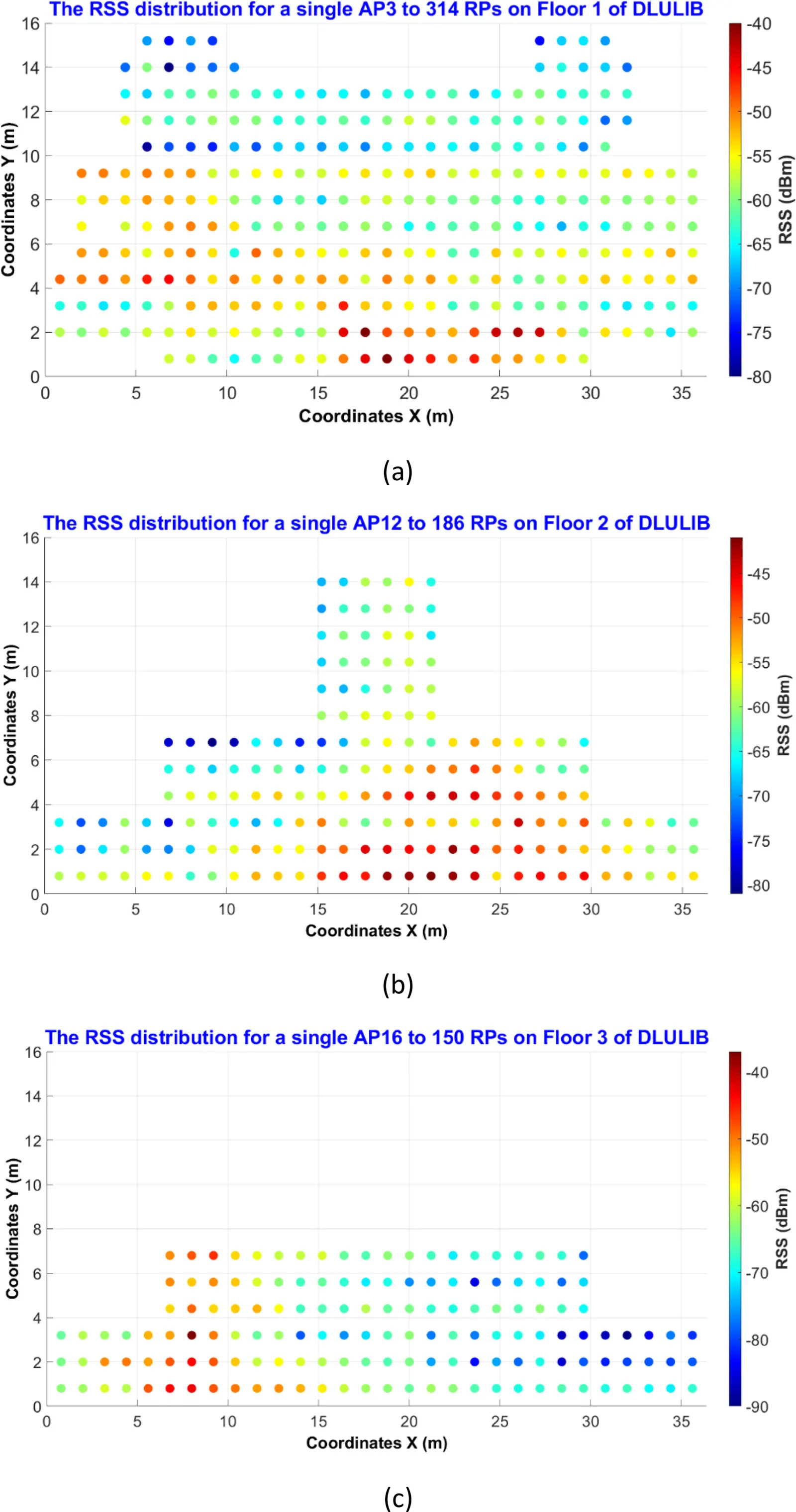
The RSS distribution for a single AP over the RPs on (a) Floor 1, (b) Floor 2, and (c) Floor 3.

Table 4 shows the number of participants and smartphone models that participated in the data collection process across the three floors. In the online phase, RSS values were collected again at the same RPs’ locations to create the testing database. At each TP, RSS values were gathered 10 times, with varying participant-facing directions instead of a fixed orientation. The exact direction of each sample and the number of samples collected for each direction, however, were not recorded. Thus, the training and testing data were collected separately, although the TPs correspond to the same physical locations as the RPs. This design provides independent RSS fingerprints for evaluating positioning methods at the predefined RPs but does not directly assess spatial generalization to random locations. The online phase simulates practical positioning scenarios in which a user’s position is estimated using real-time observed RSS fingerprints.

**Table 4:** Summary of the number of participants and smartphones across each floor.

Floor	Number of participants	Number of smartphones
Floor 1	15	15
Floor 2	8	8
Floor 3	7	7

### 4.3 Baseline positioning methods

To demonstrate the usability of the DLULIB dataset, various baseline indoor positioning methods were implemented, including KNN, WKNN, CNN, and Transformer-based models. These methods were applied to the DLULIB dataset to determine users’ positions and then calculate positioning errors. The positioning results indicate that DLULIB can support not only conventional methods, i.e., KNN and WKNN methods, but also more complex methods, i.e., CNN and Transformer-based models. The mean positioning errors of the implemented methods for the three floors are shown in Fig. 8, where CNN achieves the best overall performance. However, the positioning performance varies across floors, with CNN and Transformer-based models showing higher errors on Floor 3 than KNN and WKNN. This difference may be related to floor-specific RSS characteristics and spatial conditions. These differences could be further investigated in future studies, while the reported results provide baseline references for researchers using the DLULIB dataset. These baseline results should be interpreted as evaluations using separately collected testing data at the predefined RPs and should not be considered a direct assessment of performance at random locations.

**Fig. 8:**
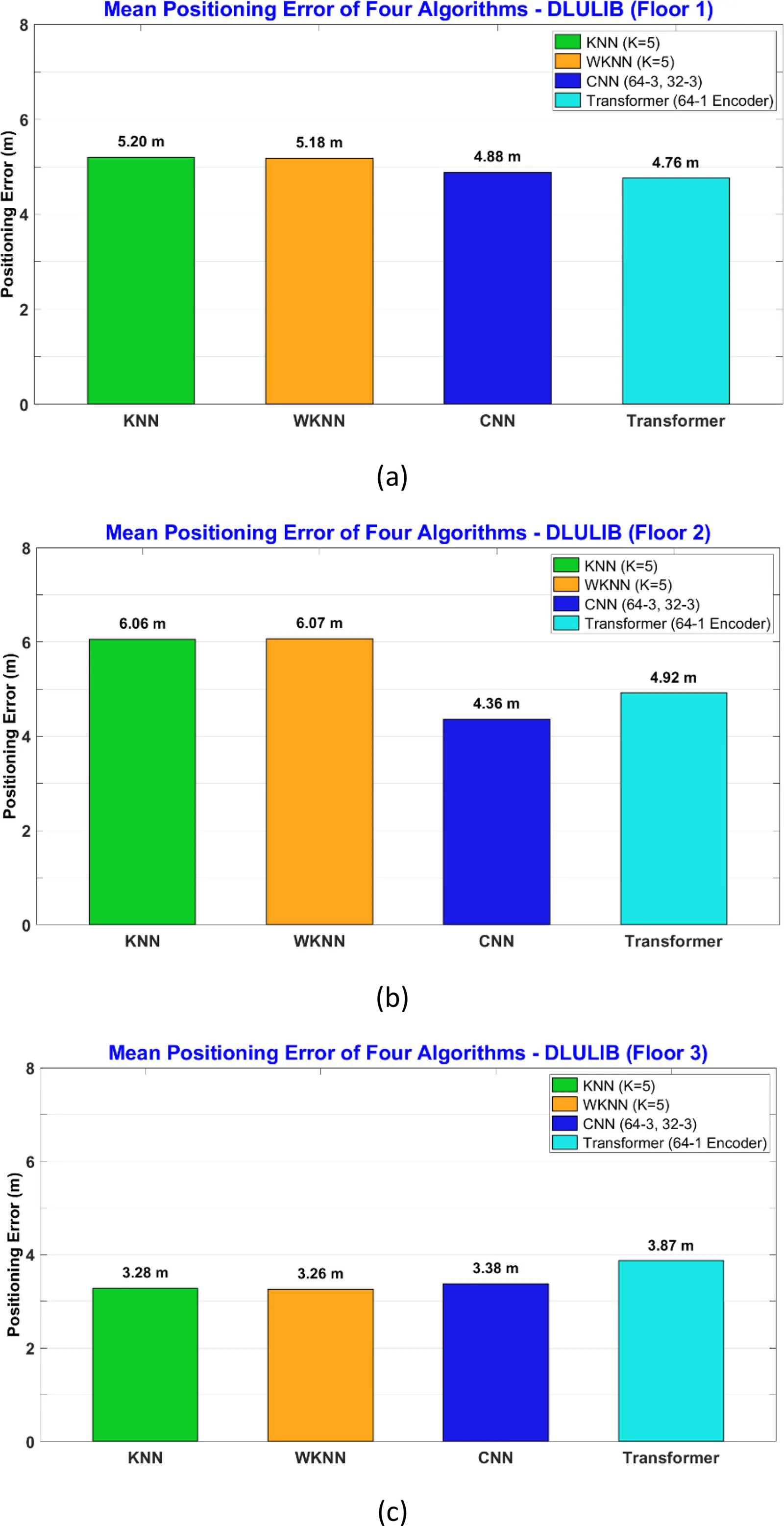
The mean positioning errors of four methods in (a) Floor 1, (b) Floor 2, and (c) Floor 3.

## Limitations

Despite the advantages, such as considering the multi-floor library buildings with complex architectural characteristics and a large number of RPs, APs, smartphones, participants, and participant-facing directions, the DLULIB dataset also faces limitations. The dataset was collected in a single multi-floor library building, which may not represent all types of buildings with different building structures, construction materials, and WiFi infrastructures. The WiFi data were collected within five months, thus, the dataset may not fully show the long-term temporal variations in RSS values caused by environmental changes, seasonal effects, or the addition/removal of APs in the positioning areas. Next, the testing data were collected separately but at the same locations as the RPs. Therefore, the dataset does not directly assess performance at random locations. Moreover, the exact participant-facing directions for individual online RSS samples were not recorded. Finally, the dataset also focuses on WiFi RSS values collected when the participants stood on one of the RPs. Thus, it does not include dynamic user trajectories, walking speeds, or continuous movement patterns that may be related to tracking-based positioning systems. Future extensions of DLULIB may include data collection across different times or seasons, spatially independent TPs, recorded online participant-facing directions, and dynamic user trajectories with different walking speeds. These extensions could support studies of temporal fingerprint stability, spatial generalization, orientation-aware positioning, and tracking-based indoor positioning.

## Credit Author Statement

**Khanh Nguyen-Huu:** Conceptualization, Methodology, Writing, Original draft preparation. **Luong Nguyen Thi:** Data curation, Visualization. **Dang Pham-Hai:** Data curation, Investigation. **Nga Thi Thanh Phan:** Software, Validation. **Trung Vu-Thanh:** Software, Investigation. **Seon-Woo Lee:** Writing- Reviewing and Editing, Supervision. **Ninh Duong-Bao:** Validation, Supervision.

## Ethics Statement

The authors have read and followed the ethical requirements for publication in Data in Brief and confirm that the current work does not involve human subjects, animal experiments, or any data collected from social media platforms.

## Data Availability

The DLULIB dataset, including the raw and processed WiFi data, training and testing subsets, baseline implementation code, and Android data collection application, is publicly available on Zenodo at: https://zenodo.org/records/19434712. Moreover, the source code of the Android application used for the data collection process is also publicly available on GitHub at: https://github.com/vvuthanhtrung/DLULIB_Data_Collection_App.

## Appendix Supplementary Materials

Image, application 1

Image, application 2
